# Release of Membrane-Bound Vesicles and Inhibition of Tumor Cell Adhesion by the Peptide Neopetrosiamide A

**DOI:** 10.1371/journal.pone.0010836

**Published:** 2010-05-26

**Authors:** Pamela Austin, Markus Heller, David E. Williams, Lawrence P. McIntosh, A. Wayne Vogl, Leonard J. Foster, Raymond J. Andersen, Michel Roberge, Calvin D. Roskelley

**Affiliations:** 1 Department of Cellular and Physiological Sciences, University of British Columbia, Vancouver, British Columbia, Canada; 2 Department of Biochemistry and Molecular Biology, University of British Columbia, Vancouver, British Columbia, Canada; 3 Department of Chemistry, University of British Columbia, Vancouver, British Columbia, Canada; 4 Department of Earth and Ocean Sciences, University of British Columbia, Vancouver, British Columbia, Canada; 5 The Program in Breast Cancer Metastasis, University of British Columbia, Vancouver, British Columbia, Canada; Dana-Farber Cancer Institute, United States of America

## Abstract

**Background:**

Neopetrosiamide A (NeoA) is a 28-amino acid tricyclic peptide originally isolated from a marine sponge as a tumor cell invasion inhibitor whose mechanism of action is unknown.

**Methodology/Principal Findings:**

We show that NeoA reversibly inhibits tumor cell adhesion, disassembles focal adhesions in pre-attached cells, and decreases the level of β1 integrin subunits on the cell surface. NeoA also induces the formation of dynamic, membrane-bound protrusions on the surface of treated cells and the release of membrane-bound vesicles into the culture medium. Proteomic analysis indicates that the vesicles contain EGF and transferrin receptors as well as a number of proteins involved in adhesion and migration including: β1 integrin and numerous α integrin subunits; actin and actin-binding proteins such as cofilin, moesin and myosin 1C; and membrane modulating eps15 homology domain (EHD) proteins. Surface labeling, trafficking inhibition, and real-time imaging experiments all suggest that β1 integrin-containing vesicles are released directly from NeoA-induced cell surface protrusions rather than from vesicles generated intracellularly. The biological activity of NeoA is dependent on its disulfide bond pattern and NMR spectroscopy indicates that the peptide is globular with a continuous ridge of hydrophobic groups flanked by charged amino acid residues that could facilitate a simultaneous interaction with lipids and proteins in the membrane.

**Conclusions/Significance:**

NeoA is an anti-adhesive peptide that decreases cell surface integrin levels through a novel, yet to be elucidated, mechanism that involves the release of adhesion molecule-containing vesicles from the cell surface.

## Introduction

The ability of tumor cells to interact dynamically with the extracellular matrix (ECM) is an important driver of invasion and metastasis [1]. A number of compounds have been developed that block the ability of integrins to bind to their cognate ECM ligands, including peptides based on the Arg-Gly-Asp (RGD) sequence found within many ECM proteins [2], [3] and integrin-blocking antibodies [4]. Other compounds block intracellular signaling events controlled by integrins [5] or metalloproteinase-mediated ECM destruction, an approach that, unfortunately, has not yet proven clinically effective [6], [7], [8]. Phenotypic cell-based screens have led to the identification of structurally diverse inhibitors of cancer cell invasion [9] and we developed a positive readout screen of this type that eliminates actutely cytotoxic compounds [10] to identify the motuporamines [10], [11], strongylophorine-26 [12], [13], avinosol [14] and the neopetrosiamides [15].

Herein, we characterize the cellular mode of action of the 28-amino acid marine sponge-derived peptide neopetrosiamide A (NeoA) [15]. In addition to inhibiting tumor cell invasion, NeoA also inhibits tumor cell adhesion to rigid ECM substrata and it causes pre-attached cells to round up and retract from the substratum. The latter retraction is associated with dissolution of focal adhesions and a decrease in cell surface β1 integrins. NeoA has three intrachain disulphide bridges that contribute to the generation of its compact ellipsoid shape with an amphipathic distribution of outwardly directed polar and hydrophobic residues which suggests that it may interact with membranes. Indeed, NeoA also triggers the rapid formation of membranous protrusions on the cell surface and the release of membrane-bound vesicles that contain a number of integrin subunits, adhesion-associated proteins, and growth factor receptors. Importantly, these effects of NeoA are rapidly induced, within minutes, before any cytotoxicity can be observed, and they are reversible upon peptide removal.

## Results

### Inhibition of cell invasion by NeoA

**Figure 1:**
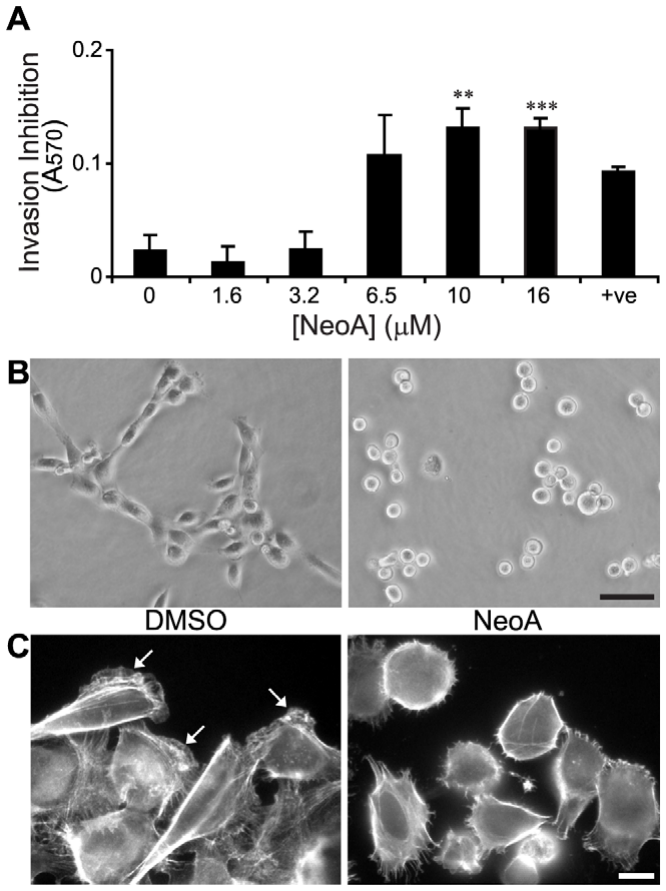
NeoA prevents tumor cell elongation and invasion into Matrigel. (A) MDA-MB-231 cells were treated with NeoA (or 5 µM dihydromotuporamine C [10] as a positive control, +ve). Live cells that failed to invade were recovered and quantified through an MTT assay. Shown are averages of triplicates ± SD. ** *P*<0.005, *** *P*<0.0005 compared to 0 µM as determined by two-tailed Student's *t*-test. (B) MDA-MB-231 cells were plated on the reconstituted basement membrane substratum Matrigel in the presence of DMSO vehicle alone or 6.5 µM NeoA and morphology was assessed by live phase contrast microscopy after 2.5 h. Scale bar, 50 µm. (C) NeoA treatment results in loss of polarity and decreased actin ruffling. f-actin staining of cells treated with either DMSO or 3.2 µM NeoA for 90 min. Note that f-actin-rich leading lamellae (white arrows in DMSO-treated cells) are greatly reduced in the NeoA-treated cells. Scale bar, 10 µm.

We initially reported that NeoA inhibited the invasion of LS174T colon carcinoma cells that move through reconstituted basement membrane gels in an amoeboid fashion [15]. Here we show that NeoA also inhibits the mesenchymal (i.e. elongated cell) invasion of both MDA-MB-231 breast carcinoma (Figure 1) and HT-1080 fibrosarcoma cells (Figure S1). The inhibition was quantified in a positive readout assay for live, non-invasive cells that we developed and validated previously [10]. Specifically, we found that NeoA inhibited approximately 3–6 times as many MDA-MB-231 and HT-1080 cells from invading into basement membrane gels at concentrations of 6.5 to 16 µM compared to DMSO-treated controls (Figure 1A, Figure S1A). Morphologically, this inhibition was associated with a rounding of a vast majority of both cell types on top of the gel (Figure 1B, Figure S1B). Furthermore, when added to tumor cells that were first allowed to pre-spread and begin migrating on rigid substrata, NeoA induced an active cellular retraction and rounding that was associated with the loss of f-actin-containing leading lamellae (Figure 1C, Figure S1C).

### NeoA disrupts focal adhesions and reversibly reduces tumor cell adhesion

**Figure 2:**
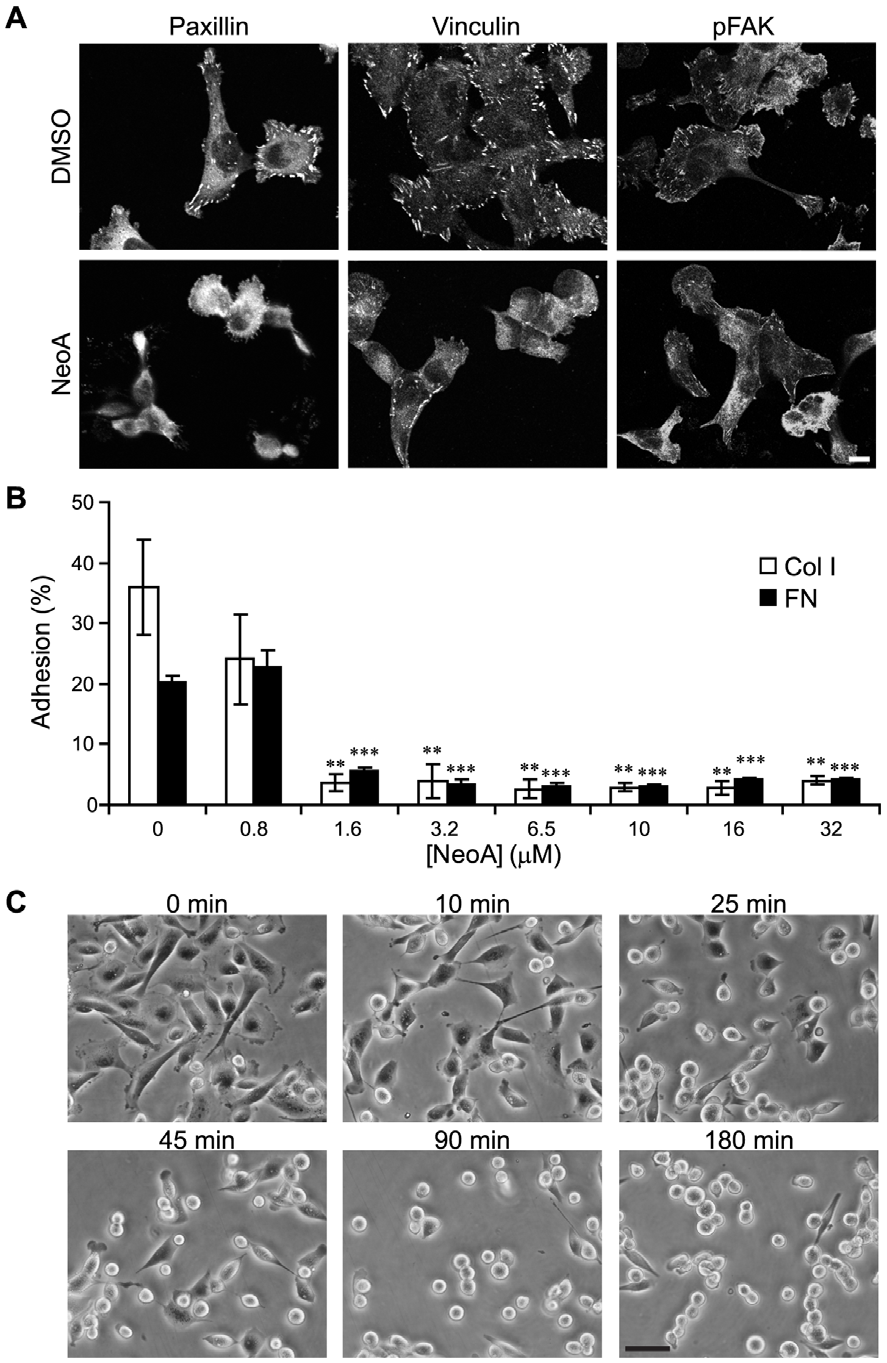
NeoA inhibits cellular adhesion and causes the disassembly of focal adhesions in MDA-MB-231 cells. (A) Cells treated for 1 h with DMSO or 6.5 µM NeoA were fluorescently stained for the focal adhesion proteins paxillin, vinculin, and phosphorylated focal adhesion kinase (pFAK). Note that all proteins were no longer localized at discrete attachment sites (i.e. focal adhesions) in NeoA-treated cells. Scale bar, 10 µm. (B) Adhesion to fibronectin (FN) and collagen type I (Col I) is significantly decreased in the presence of NeoA. Shown are averages of triplicates ± SD. ** *P*<0.005, *** *P*<0.0005 compared to 0 µM as determined by two-tailed Student's *t*-test. (C) Cells pre-attached on fibronectin begin to lose adherence between 10 to 25 min after the start of treatment with 6.5 µM NeoA. Scale bar, 50 µm.

The ability of NeoA to induce the rounding of pre-spread cells on a rigid glass substratum was associated with a disruption of focal adhesions. Specifically, NeoA treatment led to a delocalization of the focal adhesion scaffolding proteins paxillin and vinculin as well as a decrease in the phosphorylated form of focal adhesion kinase from characteristic longitudinal streaks at the cell-substratum interface in MDA-MB-231 tumor cells (Figure 2A). These observations suggested to us that NeoA may decrease cell adhesion, which was confirmed by quantifying the dose-dependent inhibition of MDA-MB-231 cell attachment to both collagen type I and fibronectin (Figure 2B). To better assess the kinetics of this anti-adhesive effect, cells were pre-spread on fibronectin-coated dishes, treated with 6.5 µM NeoA, and observed live for 90 min by phase microscopy (Figure 2C). A clear anti-adhesive rounding response was observable within 25 minutes and it was very striking after 45 minutes. All of these anti-adhesive effects were also observed in HT-1080 tumor cells (Figure S2).

**Figure 3:**
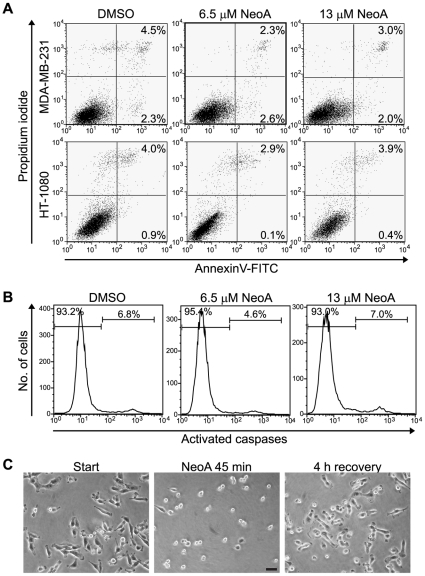
NeoA is not toxic to cells at concentrations that inhibit invasion. (A) Cell death and apoptosis of MDA-MB-231 and HT-1080 cells treated with the indicated concentrations of NeoA for 45 min were determined by assessing propidium iodide uptake (y axis) and Annexin V staining (x axis) by flow cytometry. Cells undergoing apoptosis stain positively for Annexin V-FITC alone and would appear in the lower right quadrant of the plots. Dead cells are PI-positive and appear in the upper quadrants, either without having undergone apoptosis (upper left quadrant) or after having undergone apoptosis (upper right quadrant). Note that NeoA did not increase the number of dead cells of either type compared to the negative DMSO control treatment. (B) Activated caspase levels were measured using a fluorescence-based kit and levels were assessed in MDA-MB-231 cells by flow cytometry following a 45 min treatment with NeoA at the indicated concentrations. (C) Morphological reversion following the removal of NeoA. MDA-MB-231 cells on tissue culture plastic spread prior to treatment; round after a 45 min treatment with 13 µM NeoA; and re-spread 4 h after the removal of NeoA. Scale bar, 50 µm.

MDA-MB-231 tumor cells treated with 6.5 or 13 µM NeoA for 45 min did not display an increase in Annexin V staining, propidium iodide uptake or activated caspase levels (Figures 3A, 3B). Furthermore, the anti-adhesive cell rounding and retraction caused by 45 minutes of NeoA treatment was fully reversed 4 hours after compound removal (Figure 3C). Similar results were observed with HT-1080 cells (Figure 3A, and not shown). Thus, the initial anti-adhesive effects of NeoA were not likely caused by cytotoxicity, which was expected given that our initial anti-invasion screen was designed to detect increases in viable, non-invasive cells after three hours of compound treatment (10, 15). It is important to point out, however, that there was an increase in apoptosis when cells were treated much longer than studied in detail here (i.e. for 24 hours see Figure S3). Thus, longterm NeoA treatment does induce a degree of cytotoxicity.

### NeoA decreases β1 integrin levels

**Figure 4:**
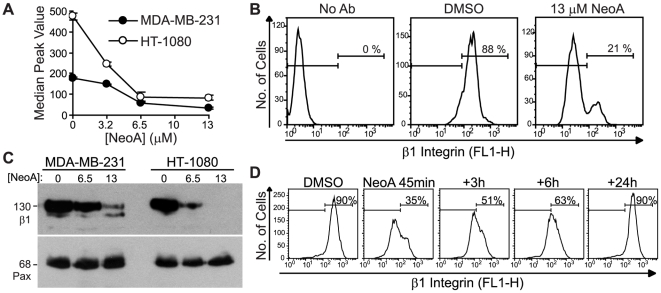
NeoA reversibly reduces levels of β1 integrin on the cell surface. (A–B) MDA-MB-231 and HT-1080 cells treated in suspension with NeoA for 45 min were stained with a FITC-labeled antibody for total β1 integrin and surface levels were evaluated by flow cytometry. (A) Graphical representation of the shift in the median peak values (averaged from duplicates within one experiment ± SD) of β1 integrin after treatment with the NeoA. (B) NeoA lowers the percentage of MDA-MB-231 cells expressing β1 integrins on their surface. (C) Immunoblots of whole cell lysates from cells treated with 0, 6.5, or 13 µM of NeoA for 45 min. A representative blot from three independent experiments was probed for β1 integrin (β1) and paxillin (Pax). (D) Decrease in β1 integrin is reversible. MDA-MB-231 cells treated for 45 min with 13 µM NeoA were washed and allowed to recover for the indicated times. Surface levels of β1 integrin were measured by flow cytometry. All data shown are representative from at least three independent experiments.

Based on the loss of cellular adhesion caused by NeoA, we reasoned that the ability of integrins to interact with ECM ligands could be affected. Indeed, flow cytometric analysis indicated that NeoA treatment caused a concentration-dependent decrease in the median peak cell surface levels of β1 integrin subunits, which are prominent in both MDA-MB-231 and HT-1080 cells (Figure 4A). This was reflected by a significant decrease in the percentage of cells with high levels of β1 integrin on their surface. Specifically, 13 µM NeoA caused a decrease from 88% to 21% of MDA-MB-231 cells showing considerable levels of β1 integrin on the cell surface (Figure 4B). Immunoblotting of whole cell lysates also showed that NeoA induced a large decrease in total cellular β1 integrin (Figure 4C). Importantly, NeoA treatment did not affect the total levels of the focal adhesion scaffolding protein paxillin (Figure 4C) despite the fact that paxillin did become diffuse and delocalized after NeoA-mediated focal adhesion disruption (see Figure 2A above).

The NeoA-induced decrease in β1 integrin on the cell surface occurred very rapidly. It was first noticeable within 20–30 minutes (not shown) and it was clearly evident after 45 minutes of treatment (Figure 4), a time frame that was not associated with any increase in cytotoxicity (see Figures 3A, 3B above). Additionally, like the morphologic response, the loss of β1 integrin subunits from the cell surface was fully reversible after compound withdrawal (Figure 4D).

### NeoA triggers the release of intact β1 integrin subunits from the cell surface

**Figure 5:**
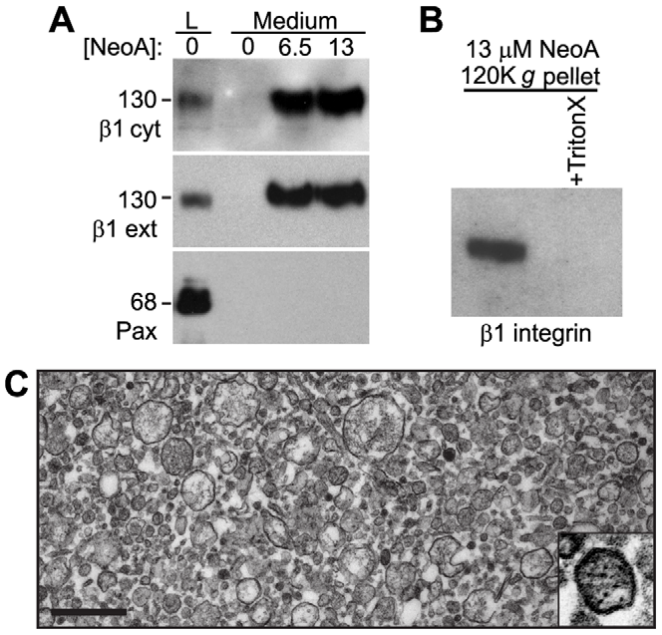
NeoA causes the release of intact β1 integrin subunits and membrane-bound vesicles. (A) Immunoblots of 10X concentrated conditioned medium from MDA-MB-231 cells treated with 0, 6.5, or 13 µM of NeoA for 45 min compared to whole cell lysates (L) of control cells treated with DMSO alone. Blots were probed using two distinct β1 integrin antibodies, one recognizing the cytosolic region (β1 cyt) and one recognizing the extracellular region (β1 ext) of the protein, as well as with an antibody for paxillin (Pax). (B) The released β1 integrin is associated with a detergent-soluble membrane bound vesicle. Conditioned medium was collected from cells treated with NeoA and one sample was pre-incubated with 1% Triton X-100 prior to ultracentrifugation. Pellets were then immunoblotted for β1 integrin. (C) TEM of vesicles from the 120,000 *g* pellet from the conditioned medium of NeoA treated cells. Scale bar, 0.5 µm.

To determine if NeoA induced the release of β1 integrin subunits from the cell surface, we examined the tissue culture medium after treatment. Indeed, immunoblotting showed that NeoA caused tumor cells to release large amounts of β1 integrin into the medium (Figure 5A). The released β1 integrin had an electrophoretic mobility of approximately 130 kDa, which is the size of the intact protein in untreated whole cell lysates (lane ‘L’ in Figure 5A). To verify that β1 integrin subunits were being released in intact form, we demonstrated that the 130 kDa protein was recognized by antibodies directed against both the extracellular and the cytosolic domains of the protein (Figure 5A). In comparison, NeoA treatment caused no discernible release of paxillin (Figure 5A). The selective release of full-length β1 integrin subunits, the lack of toxicity within the time frame of the release (within 45 minutes), and the recovery of cellular β1 integrin levels after NeoA withdrawal all suggested to us that this release was not a consequence of cell lysis.

**Figure 6:**
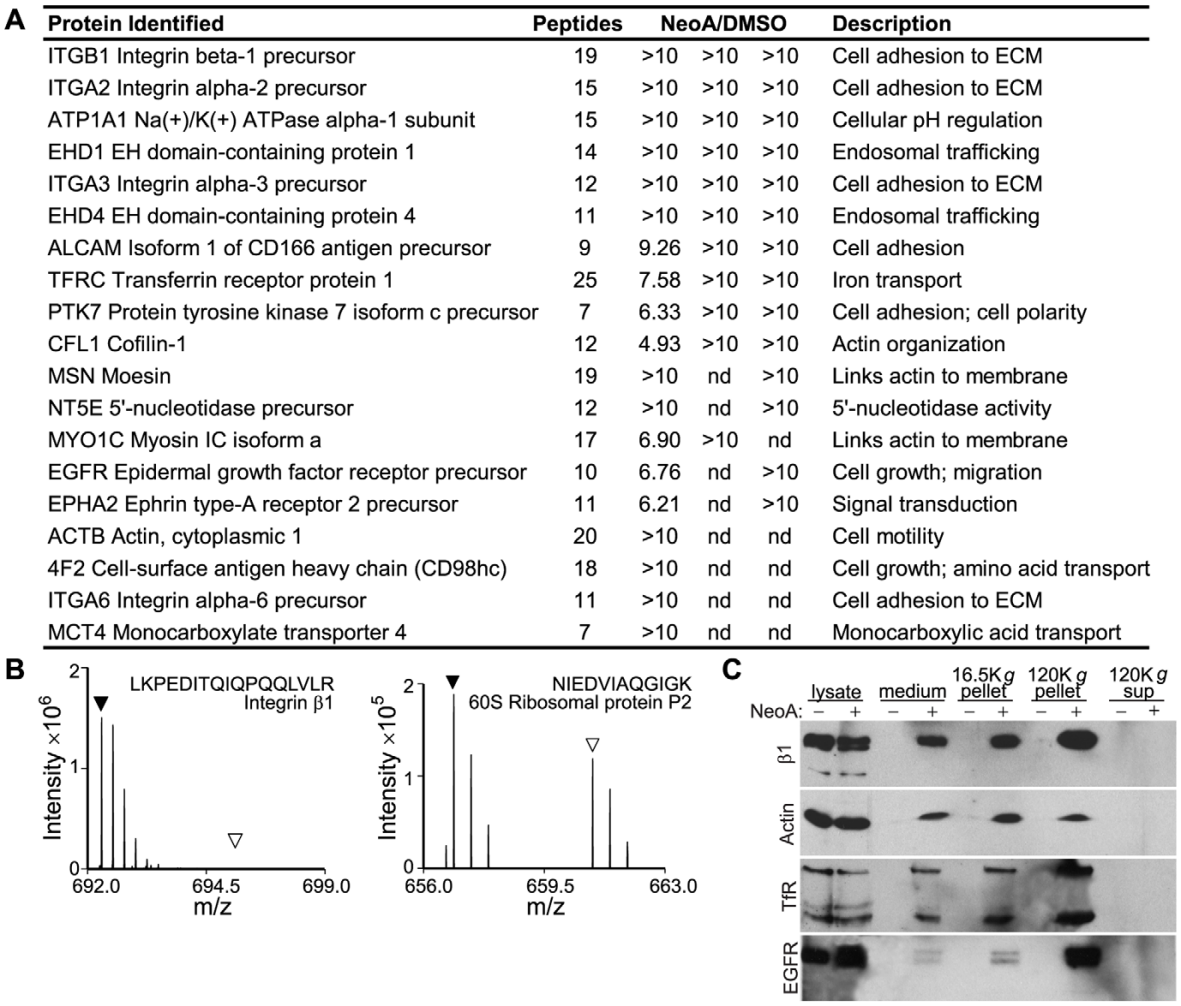
Characterization of released vesicles. (A) List of the most abundant proteins (based on spectra counts) found preferentially in the NeoA-treated pellets versus DMSO-treated pellets from MDA-MB-231 cells. Ratios of peptides in NeoA vs. DMSO pellets (NeoA/DMSO) from each of three experimental replicates analyzed are listed. nd, not detected. (B) Representative mass spectra from proteomic analysis of the pellets from NeoA-treated (black triangles) and DMSO-treated (open triangles) cells. Triangles mark the position expected for the peptide. Spectra of a protein highly enriched in the pellet of NeoA-treated cells (left) and of a protein present at similar levels in the pellets from both NeoA-treated and DMSO-treated cells (right) are shown. (C) β1 integrin (β1), actin, transferrin receptor (TfR), and EGF receptor (EGFR) can all be detected in the conditioned medium from MDA-MB-231 cells treated with 13 µM NeoA and can be pelleted at high centrifugal speeds. On the gel, from DMSO (−) or NeoA (+) treated conditions: 5 µg of cell lysate; 50 µl of conditioned medium; 1/4 of pellet from 16,500 *g* spin; 1/4 of pellet from 120,000 *g* spin; 50 µl of supernatant from final spin. Note that, since pellets in this experiment were not re-suspended in the same volume as the supernatant they were pelleted from, comparisons of protein levels on this blot can only be made between the treated and control samples for each pellet or supernatant.

Pre-treatment of tumor cells with a battery of protease inhibitors had no effect on the magnitude of the release or the molecular weight of the released β1 integrin subunits after NeoA treatment (data not shown). Thus, we next considered the possibility that β1 integrins were being released in vesicles. Following a general procedure for isolation of small vesicles, the medium of DMSO- or NeoA-treated cells was subjected to sequential centrifugations, ending in a 120,000 *g* ultracentrifugation step; samples of the supernatants and pellets from the various steps in this procedure were then immunoblotted. β1 integrin was detected only in the pellets of the samples from NeoA-treated cells but not from the DMSO-treated cells (see Figure 6C). Furthermore, pre-incubation of the medium from NeoA-treated cells with 1% Triton X-100 prior to centrifugation prevented the detection of β1 integrin in the NeoA-induced pellet (Figure 5B), suggesting it was associating with membranous vesicles. This was confirmed by imaging the pellet from the medium of NeoA-treated cells using transmission electron microscopy (TEM). While no discernible material was generated from the conditioned medium of DMSO-treated cells (not shown), the pellets generated from the conditioned medium of NeoA-treated cells were composed of membrane-bound vesicles with a mean diameter of 71 nm (±44.5 nm, SD, n = 177; Figure 5C). Actin microfilaments (approximately 6 nm cross-sectional diameter) were observable within the vesicles (inset, Figure 5C) and there was no evidence of cellular organelles or debris amongst the vesicle population.

### Biochemical characterization of the vesicles released by NeoA treatment

To determine what cellular proteins were present in NeoA-generated membrane-bound vesicles, we carried out a quantitative proteomic analysis. Little protein was detected in the pellets generated from the ultracentrifugation of conditioned medium from vehicle control (DMSO)-treated cells. In contrast, the NeoA-induced pellet contained a number of transmembrane proteins that included endogenous EGFR and the transferrin receptor (Figure 6A). The β1 integrin subunit and the α2, α3 and α6 integrin subunits were also prominent in the NeoA pellet. In addition to actin, which was expected based on the TEM, a number of actin-binding and membrane-modifying proteins that have been implicated in cell adhesion, cell migration and cell surface specializations were also present. These included moesin, cofilin, EphA2, myosin 1C and EHD proteins (Figure 6A). Representative mass spectra from the proteomic analyses are shown (Figure 6B).

To verify the proteomic results, conditioned medium was subjected to sequential centrifugations, ending in a 120,000 *g* ultracentrifugation. Samples of the supernatants and pellets from the various steps in this procedure were then immunoblotted and β1 integrin, actin, transferrin receptor, and EGFR were all detected in the conditioned medium from NeoA-treated cells and in the pellets (i.e. the vesicle-containing fraction) of the samples from the NeoA-treated, but not from the vehicle control, DMSO-treated, cells (Figure 6C).

### NeoA triggers the formation of membrane projections and the release of membrane-bound vesicles from the cell surface

**Figure 7:**
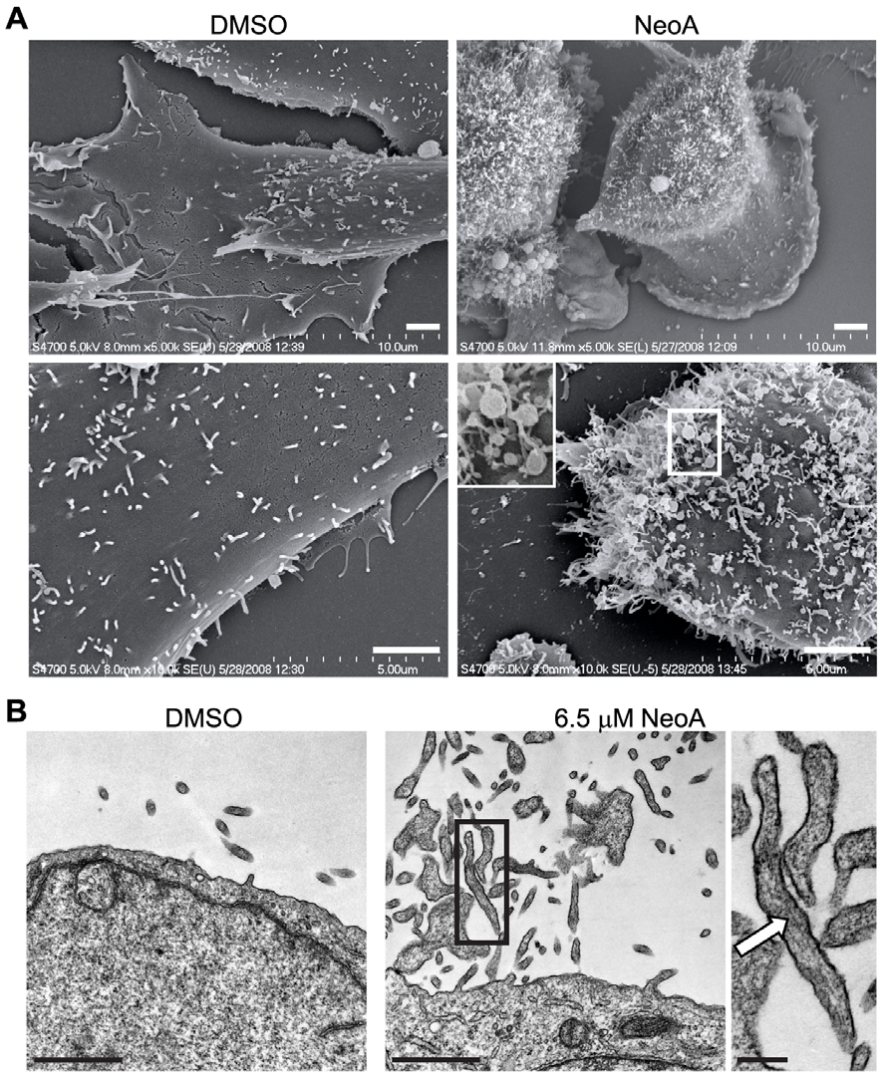
NeoA induces formation of membrane-bound protrusions. (A) SEM of the cell surface reveals an increase in membrane projections upon treatment with NeoA. MDA-MB-231 cells treated with DMSO (left panels) or 6.5 µM NeoA (right panels) for 30 min. Different cells are shown from two magnifications. Scale bars, 2 µm. (B) TEM confirms the presence of membrane protrusions at the apical cell surface. Left panel: cells treated with DMSO. Middle panel: cells treated for 20 min with 6.5 µM NeoA. Right panel is a higher magnification view of inset outlined by the black box in the middle panel; Actin filaments indicated by arrow. Scale bars in left and middle panels, 1 µm; in right panel, 0.2 µm.

To further understand the release of vesicles from NeoA-treated cells, we examined the cell surface by scanning and transmission electron microscopy (SEM and TEM, respectively). SEM indicated that DMSO/vehicle control-treated MDA-MB-231 cells plated on serum-coated coverslips were flat and had small, somewhat sparse, microvilli on their dorsal surface (Figure 7A). Within 30 minutes, NeoA caused the cells to round up and, strikingly, it induced the formation of numerous irregularly shaped protrusions on the cell surface that were often terminally distended (inset Figure 7A). TEM indicated that these projections were membrane-limited and contained filamentous actin (arrow, right panel inset; Figure 7B). TEM also indicated that the plasma membrane between the NeoA-induced projections was not damaged or denuded. In addition, there was no observed increase in subplasmalemmal vesicles or multivesicular bodies which suggests that NeoA did not substantially increase intracellular vesicle trafficking.

### NeoA-induced membrane protrusions are highly dynamic and release transmembrane protein-containing vesicles

The EGFR is a prominent transmembrane protein in MDA-MB-231 cells [16] and was shown to be released in NeoA-induced vesicles (Figure 6A,C). Therefore, we expressed an EGFR-YFP fusion protein in these cells by transient transfection to mark the plasma membrane and then imaged the cells, live, by spinning disk confocal microscopy.

**Figure 8:**
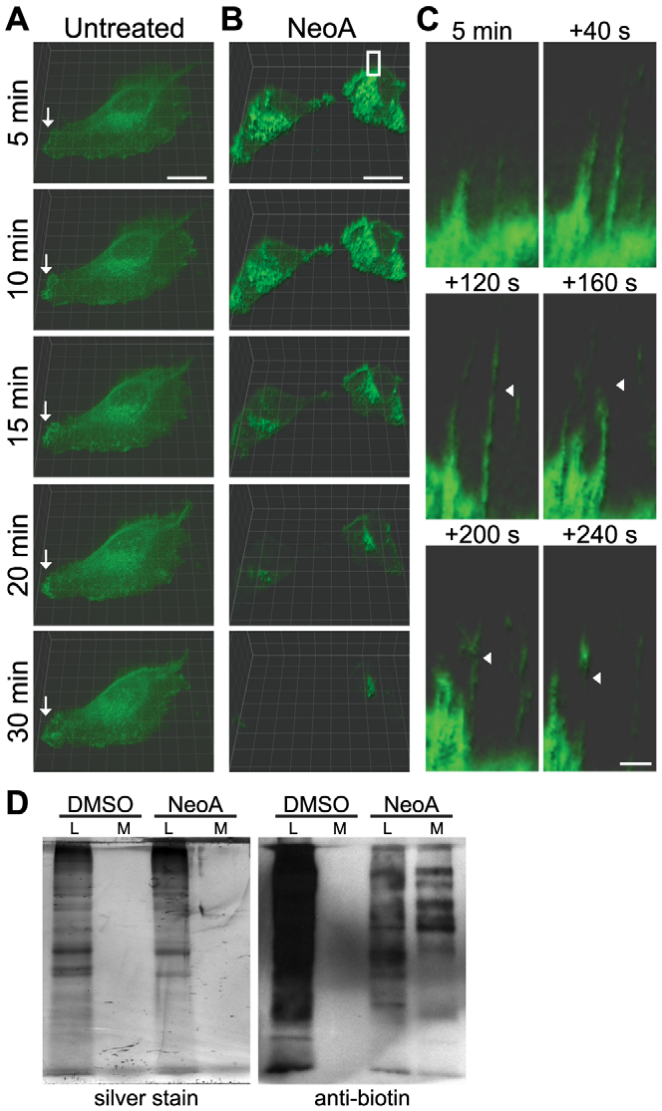
Vesicles are released from the cell membrane of NeoA-treated cells. (A–C) Live cell images of MDA-MB-231 cells transiently transfected with EGFR-YFP at several time points throughout a 30 minute treatment. Shown are representative cells out of at least 20 cells imaged in 3 separate experiments. (A) Untreated cells. Arrows point to areas of EGFR at membrane ruffles. (B) Cells treated with 6.5 µM NeoA. Scale bar, 10 µm. (C) Higher magnification view of surface projections from a NeoA-treated cell marked by the white box in panel B. After an initial 5 min treatment period the projections were observed for an additional 240 seconds. Arrowheads point to areas where vesicles appear to be pinching off from the protrusions. Scale bar, 1 µm. (D) Cell surface proteins were biotinylated prior to treatment with DMSO or 13 µM NeoA for 45 min at 37°C. Following treatment, cell lysates (L) and medium (M) were collected. Equal proportions (1/5 of total sample from the same cell numbers) were run on SDS-PAGE. One gel was silver stained to indicate total protein(s) while one was transferred and probed with anti-biotin to indicate proteins that were originally on the cell surface.

In untreated cells, membranous EGFR-YFP was distributed across much of the cell surface with increased accumulations over the cell body and at areas of membrane ruffling (arrows, Figure 8A). Overall there were no striking changes in EGFR-YFP distribution over a 30 minute observation period of the untreated cells. Additionally, no decrease in fluorescence intensity was observed, which indicated a lack of photobleaching over the 30 minute observation period.

Cells began to round and displayed numerous EGFR-YFP-rich projections within 5 minutes of NeoA addition (Figure 8B). Subsequently, over the ensuing 25 minutes, much of the EGFR-YFP intensity was lost from the cell surface (Figure 8B). Upon examination of individual cell surface projections at higher magnification over a four minute (240 second) period beginning five minutes after the addition of NeoA, the projections were observed to be very dynamic and many of them appeared to directly release small, discrete EGFR-YFP labeled vesicle-like structures as shown by the arrowheads in Figure 8C.

In an effort to further assess if vesicles are released directly from NeoA-induced protrusions, we biotinylated cell surface proteins prior to treatment and compared cell surface protein levels in cellular lysates and in the vesicular fractions released in the media. In untreated controls, the majority of surface-labeled proteins were present in the cell lysate (Figure 8D). In contrast, in the NeoA-treated condition, the overall abundance of biotinylated proteins was reduced in the cell lysate despite the fact that total protein levels were similar between the untreated and NeoA-treated cell lysates (compare silver stains and the anti-biotin westerns of the lysate fractions in Figure 8D). In addition, there was a clear and notable increase in the number of biotinylated protein species in the medium/vesicular fraction of the NeoA-treated cells. The biotinylated band patterns of the cell lysates and medium/vesicular fractions recovered from NeoA-treated cells were similar but not identical, suggesting that cell surface proteins were not all released equally.

### The activity of NeoA is temperature-sensitive and dependent on its compact and distinct amphipathic structure

**Figure 9:**
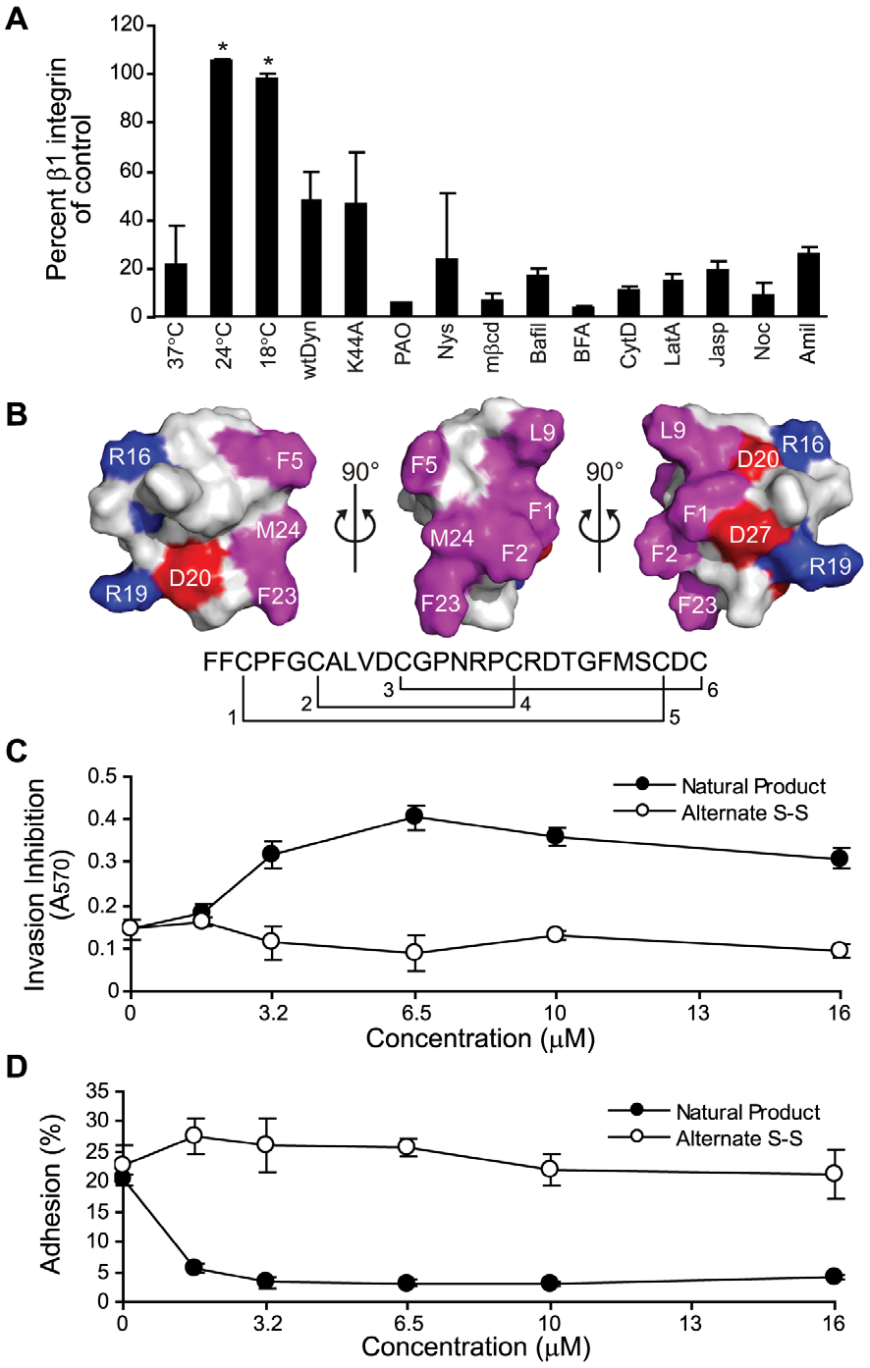
NeoA's effects are temperature sensitive and structure dependent. (A) Percent β1 integrin levels (compared to untreated vehicle controls) on the cell surface of NeoA-treated cells under the indicated temperature conditions (i.e. 37°C or 24°C), or in the presence of the following inhibitors all at 37°C: wild-type dynamin control (wtDyn); dominant negative dynamin (K44A); phenylarsine oxide (PAO); nystatin (nys); methyl-β-cyclodextrin (mβcd); bafilomycin (Bafil); brefeldin A (BFA); cytochalasin D (CytD); latrunculin A (LatA); jasplakinolide (Jasp); nocodazole (Noc); or amiloride (Amil). Surface β1 integrin levels were measured by flow cytometry. Results are averages from two independent experiments. * *P*<0.05 compared to cells treated at 37°C as determined by two-tailed Student's *t*-test. (B) Surface representation of NeoA, with hydrophobic residues shown in magenta, whereas positively- and negatively-charged residues are in blue (Arg) and red (Asp), respectively. Note the large hydrophobic patch flanked by charged groups. MDA-MB-231 cells were assayed for (C) invasion and (D) adhesion after treatment with the natural NeoA peptide (Natural product) or a synthetic peptide with an alternate disulfide bond connectivity (Alternate S-S). Shown are averages of triplicates ± SD.

In an effort to gain further insight into the mechanisms by which NeoA induces a reduction in β1 integrins and vesicle release from the cell surface, we attempted to block this decrease by various means. Cells infected with a specific inhibitor of endocytosis (a dominant negative K44A dynamin mutant) or treated with pharmacologic endocytosis inhibitors (phenylarsine oxide, nystatin, methyl-β-cyclodextrin) still lost considerable β1 integrin after NeoA treatment (Figure 9A) and they continued to release NeoA-induced vesicles (assessed with a Nicomp particle sizer; data not shown). Additionally, inhibitors of intracellular trafficking (bafilomycin A1, brefeldin A), actin disruptors (cytochalasin D, latrunculin A, jasplakinolide), a microtubule disruptor (nocodazole), and an inhibitor of macropinocytosis (amiloride) all had little or no effect on NeoA-induced loss of β1 integrin. The only effective blockade of NeoA-mediated integrin-release occurred when the incubation temperatures were reduced to either 24°C or 18°C (Figure 9A). This suggested to us that NeoA induces vesicle release via a mechanism requiring plasma membrane fluidity.

NeoA consists of 28 standard amino acids, except for a methionine sulfoxide at position 24, and is a tricyclic peptide with three disulfide bridges [15]. Using NMR-derived distance and dihedral angle restraints, we have now determined the three-dimensional structural ensemble of NeoA. The peptide folds into a compact, oblate ellipsoid shape. A summary of the restraints and structural statistics for this ensemble is provided (Table S1). Although it follows hairpin-like arrangements, the backbone of NeoA does not exhibit any regular secondary structure with the exception of residues centered around Arg19 and Asp27 that are aligned in an anti-parallel β-strand fashion. However, tertiary folding leads to a distinct amphipathic distribution of polar and hydrophobic residues. Specifically, with the exception of Phe5, all hydrophobic groups form a contiguous ridge with the side chain of the atypical, oxidized methionine (Mso24) located between the aromatic rings of Phe2, Phe5, and Phe23 (Figure 9B). In a similar fashion, the charged side chains of Arg16, Arg19, Asp11, Asp20, and Asp27 are localized on either side of this non-polar cluster. Thus, given its globular, amphipathic structure it is possible that NeoA can interact simultaneously with lipids and proteins in the membrane.

Very recently, Vederas and colleagues generated data indicating that the specific disulphide linkages in NeoA are: Cys3 to Cys26 (bridge position 1–5), Cys7 to Cys18 (bridge position 2–4), and Cys12 to Cys28 (bridge position 3–6) [17]. We confimed this by demonstrating that a synthetic peptide with this bridge connectivity has anti-invasive activity [17]. Initially, however, we reported that the natural form of NeoA purified from a marine sponge extract had a slightly different, ‘alternate’ bridge connectivity of 1–5, 2–3, 4–6 [15]. The residues involved in the alternate bridging (ie. Cys7, Cys12, Cys18 and Cys28) are all closely juxtaposed in the NMR-derived structure and modeling indicates that the alternate connectivity would not affect the globular amphipathic nature of the peptide's structure described above (data not shown). Despite this, a synthetic peptide containing the alternate bridge connectivity generated by the Vederas group [17] was not anti-invasive (Figure 9C), nor did it prevent cell adhesion (Figure 9D). Therefore, the biological activity of NeoA is likely very sensitive to the subtle structural changes that the alternative disulphide bridging gives rise to. This suggests that the peptide's cellular target(s) may also be structurally specific.

## Discussion

A number of structurally diverse agents that target cellular invasion, including those that inhibit the controlled invasion of endothelial cells that drives angiogenesis, act predominantly by altering the cytoskeleton. This includes the compounds fumagillin, TN-470, thrombospondin 1 and endostatin [18], as well as motuporamines and strongylophoryine-26 which were identified by our group [11], [13]. All of these compounds increase focal adhesion stability to inhibit productive cell motility [11], [13]. In contrast, NeoA caused attached and spread cells to retract and round-up as focal adhesions were dismantled. These effects were not limited to tumor cells. They also occurred when normal fibroblasts and endothelial cells were treated with NeoA (data not shown) which suggests that the peptide is generally anti-adhesive.

NeoA induces cells to form membranous protrusions with cytoplasmic cores that contain filamentous actin. These membranous protrusions are very prominent on the apical, free surface of the attached tumor cells and they have a morphology that is distinct from microvilli, which tend to be shorter (1–2 µm) and more regular in length and diameter. Microvilli also lack adhesion proteins such as integrins [19], [20] which are most often associated with the basal, attached surface of adherent cells. On the other hand, filopodial projections often contain integrins but, unlike NeoA-induced protrusions, filopodia are often attached to the substratum. NeoA-induced protrusions were long and often distended terminally or along their length. Live imaging indicated that these protrusions formed very quickly, within five minutes, were highly dynamic, and that vesicles were likely released directly from their distended portions.

The vesicles released by cells treated with NeoA contained β1 integrin subunits, α integrin subunits, actin and several proteins that have previously been shown to associate with integrins or actin. In fact, with the exception of EGFR and transferrin receptors, which were also present in the vesicles, nearly all of the 19 most prominent proteins found in the vesicles play some role in cell adhesion or motility. The three most prominent integrin α subunits detected in the NeoA vesicles can all dimerize with β1 integrin to bind ECM components. EHD1 regulates transport of β1 integrin [21] and EHD4 has been reported to be involved in binding to ECM [22]. Additionally, these two EH domain-containing proteins have been shown to interact with each other [23]. CD98hc and MCT4 associate together with other proteins in a complex [24] that also associates with β1 integrin [25], [26]. 5′-nucleotidase has been shown to promote invasion, migration and adhesion of cancer cells [27] and both CD166 and protein tyrosine kinase 7 are thought to be cell adhesion molecules [28], [29]. Actin, widely known to be involved in cell motility, is present along with cofilin-1 which binds actin [30], and moesin and myosin 1C which link actin to the plasma membrane [31], [32]. Our quantitative proteomic analysis also indicated that a number other integrins subunits, including β4, β5, α5, and αv, were also present in the vesicles, albeit to a lesser degree (data not shown). The release of these integrin subunits may explain why the peptide is also anti-adhesive for endothelial cells, given that the latter utilize αv- and β5-containing integrins to facilitate adhesion to the substratum [33]. However, it is worth noting that the decrease in β1 integrins induced by the treatment of tumor cells with NeoA also occurred when those cells were maintained in suspension (Figure 4). Thus, in future studies it will be important to determine if the peptide has integrin-independent effects on adhesion or other cellular functions.

A relatively unbiased RNAi-based screen found that genes which modulate β1 integrin and regulators of actin dynamics form two of three major nodes that regulate cell migration [34]. While NeoA did not induce the vesicular release of adhesion-modulating proteins with absolute specificity, these two functional categories of proteins were highly enriched in the vesicles. Additionally, the proteins released into the vesicles from the cell surface after biotinylation were different to those that remained on the cell surface after NeoA treatment (Figure 8D). Thus, there may be some specificity to the proteins released from cells in the vesicles that, due to their loss, could selectively impinge on cell adhesion and migration.

The effects of NeoA on adhesion occurred rapidly, within minutes, in the absence of any observable cytotoxicity, and these effects were fully reversible. However, when cells were treated continuously with NeoA for 24 hours some apoptosis did occur. The reasons for the latter cytotoxic response are not yet clear. One possibility is that longterm NeoA treatment, by virtue of its ability to dismantle focal adhesions and release cells from the substratum, may induce the anoikic state of apoptosis that often occurs when adherent cells are maintained in suspension [35]. Determining if this is, indeed, the case will require a detailed dose and kinetic response analysis wherein the ability of NeoA to induce apoptosis in anoikis-sensitive and resistant cell lines maintained either in adherent or suspension culture are compared. On the other hand, we reiterate that NeoA was able to initiate a decrease in cell surface β1 integrin and vesicle release from cells maintained in suspension. Thus, it is also possible that the cytotoxic effects of longterm NeoA treatment are caused by, for example, the large scale loss of surface membrane regardless of the state of cellular adhesion. If the latter turns out to be the case it could impact NeoA's further development as a potential therapeutic agent given that the development of adhesion-independent anoikic resistance appears to be a functionally important phenotype that may contribute to metastatic tumor progression [36].

Transmission electron micrographic analyses of NeoA-treated cells did not reveal an increase in multivesicular bodies within the cell cytoplasm near the plasma membrane as would be expected if vesicle release was exosomal in nature [37]. In addition, blocking trafficking pathways that are required for exosomal vesicle production and release from multivesicular bodies did not prevent NeoA-mediated vesicle release, and our live imaging analyses indicated that small, EGFR-decorated structures were released directly from NeoA-induced surface projections. Furthermore, we found that NeoA treatment led to a decrease in total cell volume (data not shown), which is expected if the peptide is causing the direct release of membrane-bound vesicles from the cell surface rather than exosome-based release of internally stored membrane-bound vesicles.

There are reports that serum treatment of MDA-MB-231 breast cancer cells can induce the direct shedding of vesicles from the cell surface that contain metalloproteinases (MMPs) [38]. However, there was no evidence of MMPs in our proteomic analysis of NeoA-induced vesicles and the conditioned medium from NeoA-treated MDA-MB-231 cells did not exhibit increased MMP activity by enzymatic zymography (data not shown). Therefore, the mode of NeoA action does not appear to mimic that induced by serum components that cause direct vesicle shedding from MDA-MB-231 cells. It is important to note, however, that vesicles directly shed by activated neutrophils do, like NeoA-induced vesicles, contain integrins [39]. Regardless, NeoA appears to induce vesicle shedding by an as yet uncharacterized novel mechanism.

The direct shedding of vesicles from the cell surface is now recognized as a contributor to paracrine signaling as it can facilitate the transfer of proteins and lipid between cells across extracellular spaces [40] in response to a number of different stimuli including calcium ionophore- and phorbol ester-mediated signaling [41]. Thus, while it is possible that NeoA co-opts the signaling pathways initiated by such compounds to rapidly induce vesicle shedding within minutes, it is important to note that the only way we could efficiently block NeoA-mediated vesicle shedding was to cool cells to temperatures of 24°C or lower. This suggests that NeoA is only active when plasma membranes are fluid. Interactions with fluid membranes could be facilitated by the amphipathic nature of NeoA's globular structure that may permit simultaneous interactions with lipids and proteins in the membrane. If such simultaneous interactions do occur they are likely highly specific because altering the disulfide bonding pattern of NeoA, which appears to only change its general globular amphipathic structure slightly, completely abrogates the biological activity of the peptide.

Database searching did not yield any known proteins or peptides with sequences or motifs that are similar to NeoA. Like NeoA, EGF domains contain three internal cysteine bridges and these domains, which are found in many proteins, can be anti-adhesive, particularly when they contain an integrin-binding RGD sequence [42]. However, NeoA does not contain such an RGD sequence, does not have the same disulfide bridge connectivity as EGF domains, and it lacks the prominent secondary structure (i.e. folded β-sheets) normally found in EGF domains [43]. The ‘P’ conotoxin peptides, which have neurotoxic activity and are produced by predatory snails, also have three internal cysteine bridges [44] and like NeoA, require proper disulfide connectivity for bioactivity [45]. However, ‘P’ conotoxins are composed primarily of hydrophilic and charged amino acid residues [46] which means that they are not likely to be amphipathic like NeoA. Despite our best efforts, we have been unable to determine how NeoA interacts with cells. This has proven very difficult using the unlabeled natural product compound, presumably because the interactions are transient and because attempts to label the natural product extinguished its biological activity. Very recently, Vederas and colleagues have been able to synthesize an active form of NeoA [17]. This should greatly facilitate the structural and functional analysis of NeoA and make it possible to generate labeled, active forms of the peptide that should help identify its precise cellular and molecular targets by optical, biochemical and immunological means.

## Materials and Methods

### Cell culture and reagents

Human breast carcinoma MDA-MB-231 and human fibrosarcoma HT-1080 cells were routinely maintained in monolayer culture in Dulbecco's Modified Eagle's Medium (DMEM) containing 10% fetal bovine serum (FBS; Invitrogen). All cells were grown at 37°C in a humidified 5% CO_2_ incubator. Unless otherwise noted, cells were harvested using a solution of 0.5 g/l of trypsin and 0.2 g/l of EDTA•4Na (Invitrogen). In cases where it was important to keep all cell surface proteins intact, a solution of enzyme-free 0.2 g/l EDTA•4Na and 0.2 g/l glucose in PBS was used to dissociate the cells. Inhibitors and reagents used: 10 µM phenylarsine oxide, 25 µM nystatin, 5 mM methyl-β-cyclodextrin, 100 nM bafilomycin A1, 5 µM brefeldin A, 2 µM cytochalasin D, 2 µM latrunculin A, 10 µM nocodazole, and 3 mM amiloride, all from Sigma. Jasplakinolide was isolated from a marine sponge extract and used at 0.5 µM. At all concentrations of inhibitors tested and when treating cells with NeoA, cells were exposed to 0.5% DMSO (v:v). For labeling of cell surface proteins with biotin, EZ-Link Sulfo-NHS-SS-Biotin (Pierce) was used according to manufacturer instructions. The labeling reaction was performed at 4°C to inhibit endocytosis and ensure only cell surface proteins were labeled prior to treatment with NeoA.

### Assay for invasion inhibition

Invasion inhibition assays were performed as previously described and validated [10]. Briefly, tumor cells were plated on reconstituted basement membrane gels (Matrigel; BD Biosciences). After 2.5 h, non-invasive cells were recovered from the top surface of the Matrigel, and the number of viable cells was quantified using a 3-(4,5-dimethylthiazol-2-yl)-2,5-diphenyltetrazolium bromide (MTT) assay.

### Immunofluorescence staining

Cells were pre-spread on coverslips in serum-containing medium and maintained for 48 h. Medium containing NeoA or DMSO was then added to the cells for the specified lengths of time prior to fixation. Cells were fixed with 3% paraformaldehyde for 15 min at room temperature, permeabilized with 0.3% Triton X-100 in PBS for 15 min, and blocked with 3% bovine serum albumin (BSA; Sigma) in PBS for 30 min, followed by incubation with mouse anti-human vinculin (Sigma) at 1/50, mouse anti-human paxillin (BD Biosciences) at 1/50, or rabbit anti-FAK [pY397] (Invitrogen) at 1/100. An Alexa Fluor 568-conjugated goat anti-mouse secondary antibody (Molecular Probes) was used at a dilution of 1 in 100 and imaged using an Olympus FV1000 confocal microscope (Central Valley, PA) to obtain single optical slices from the cell-substratum interface. To visualize f-actin, cells were stained with a 165 nM solution of rhodamine-phalloidin (Invitrogen) and imaged using a Nikon Eclipse E400 epifluorescent microscope equipped with a Q Imaging microimager II camera.

### Adhesion Assay

High binding 96-well plates (Corning) were coated with 5 µg/cm^2^ of fibronectin (Sigma) or collagen type I (Cultrex) at 37°C for 1 h. Unbound matrix was removed and wells were blocked with 1 mg/ml BSA in PBS for 1 h. Cell lines were harvested using EDTA and suspended in DMEM without phenol red supplemented with 0.1 mg/ml BSA (DMEM/BSA). Cells were fluorescently labeled with 25 µM CMFDA Cell Tracker Green (Invitrogen) for 20 min, washed with DMEM/BSA, resuspended at 2.5×10^5^ cells/ml and incubated for a further 30 min at 37°C. NeoA was added to wells of the coated 96-well plate at various concentrations in a volume of 1 µl, followed by the addition of 100 µl of the cell suspension. After 45 min, a baseline reading was obtained on a Bio-Tek FL600 fluorescent plate reader with an excitation at 490 nm and emission at 520 nm. Non-adherent cells were then removed by 3 washes with DMEM/BSA. A second fluorescence reading was then obtained and adhesion was calculated as a percentage of cells remaining compared to cells present in the baseline reading.

### Apoptosis and cell viability assays

Cells cultured as attached monolayers were treated with NeoA. The conditioned medium and one PBS wash were then collected to recover any detached cells and all remaining adherent cells were detached with EDTA. All cells were then pooled, collected through centrifugation, re-suspended in 100 µl binding buffer (10 mM HEPES pH 7.4, 140 mM NaCl, 2.5 mM CaCl_2_) and incubated with 5 µl FITC Annexin V (BD Biosciences) and 10 µl of propidium iodide (PI; 50 µg/ml; Sigma) for 15 min at room temperature. Staining for PI (relative fluorescence intensity measured in FL2 channel) and Annexin V (measured in FL1 channel) was assessed by flow cytometry on a FACSCalibur instrument (BD Biosciences) followed by data analysis using FlowJo software (Tree Star Inc). DMSO served as a negative control. A CaspaTag Pan-Caspase fluorescein labeled assay kit (Chemicon) was used to measure levels of active caspases following treatment and levels were assessed by flow cytometry in the FL1 channel.

### Flow cytometric analysis of cell surface β1 integrin

Cells in suspension were incubated with NeoA for 45 min at 37°C, washed once with PBS, and re-suspended in 200 µl buffer (PBS, 20 mM glucose, 1% BSA). 2×10^5^ cells were incubated with a 1 in 10 dilution of the directly conjugated CD29-FITC pan antibody (clone K20, Immunotech) for 45 min at room temperature. Levels of bound antibody were measured by flow cytometry on a FACSCalibur instrument (BD Biosciences) followed by data analysis (and determination of median peak values) using FlowJo software (Tree Star Inc).

### Immunoblot of cell lysates and conditioned medium

Prior to initiating the experiment, cell cultures were washed 3 times with serum-free medium to remove serum proteins. Cells were then treated with either DMSO or the indicated concentration of NeoA in serum-free medium for 45 min at 37°C, after which the conditioned medium was harvested and concentrated using 10 kDa cut-off Microcon centrifugal filters (Millipore). Non-adherent cells were pelleted by centrifugation at 500 *g* for 6 min at 4°C and kept on ice. The remaining adherent cells and the centrifuged non-adherent cells were lysed in RIPA buffer containing protease inhibitors (50 mM Tris pH 7.5, 150 mM NaCl, 1% Nonidet P-40, 0.5% sodium deoxycholate, 0.1% SDS, 20 µg/ml aprotinin, 10 µg/ml leupeptin, 100 µM PMSF, 10 µg/ml pepstatin A, 2.5 mM EDTA) at 4°C for 30 min with agitation and then clarified through centrifugation at 10,000 *g* for 15 min at 4°C. All samples for electrophoresis were quantified with a BCA protein assay (Pierce) and equalized accordingly prior to electrophoresis. The following primary antibodies were used: 0.1 µg/ml mouse monoclonal anti-human β1 integrin (extracellular immunogen, BD Biosciences), 1/500 dilution of rabbit polyclonal anti-human β1 integrin (cytosolic immunogen AB1952, Chemicon), 25 ng/ml mouse anti-human paxillin (BD Biosciences), 1/500 dilution of monoclonal anti-actin (Sigma), 1 µg/ml rabbit polyclonal anti-EGFR (Santa Cruz), and 1 µg/ml mouse anti-human transferrin receptor (Zymed). To detect biotinylated protein, an anti-biotin-HRP antibody was used (Cell Signaling).

### Vesicle isolation and characterization

Confluent MDA-MB-231 monolayers in a 10 cm dish were rinsed with serum-free medium and treated with either DMSO or 13 µM NeoA in 4 ml serum-free, phenol red-free medium. After 45 minutes at 37°C, conditioned medium was collected and all further steps were conducted at 4°C. Samples were first centrifuged at 500 *g* for 10 min to eliminate cells and supernatants were sequentially centrifuged at 16,500 *g* for 20 min and 120,000 *g* for 70 min. Small aliquots of supernatants from each step were kept aside and pellets for immunoblotting were suspended in medium. For proteomic analysis, the final vesicle pellet was solubilized in 50 µl 1% sodium deoxycholate, 50 mM NH_4_HCO_3_. Protein solutions were processed as described [47] and peptides from the control- and NeoA-treated conditions were differentially labeled with formaldehyde isotopologues (Cambridge Isotopes Laboratory) and analyzed on an LTQ-Orbitrap (ThermoFisher Scientific, Bremen, Germany) exactly as described [47]. Peptides and proteins were identified from tandem mass spectra using Mascot (v2.2) to search against the human International Protein Index database (69,186 sequences) using the following search parameters: 5 parts-per-million peptide mass accuracy, 0.6 Da fragment mass accuracy, carbamidomethylation of Cys (fixed), dimethylation (normal and with ^2^H_4_) of Lys and N-termini (variable), ion trap fragmentation characteristics and trypsin cleavage specificity with up to one missed cleavage. Proteins were considered identified if at least two unique peptide sequences with IonsScores over 26 were detected.

### Electron microscopy

For TEM, sub-confluent MDA-MB-231 monolayers on 1 µm pore culture inserts (Falcon 35-3104) were treated for 20 min at 37°C and vesicle pellets were prepared as described below. Cells on cut-out inserts and vesicle pellets were fixed (1.5% paraformaldehyde, 1.5% glutaraldehyde, 0.1 M sodium cacodylate, pH 7.3) at room temperature for 3 h, placed in buffer (0.1 M sodium cacodylate, pH 7.3) overnight at room temperature, post-fixed for 1 h on ice (1∶1 mixture of 2% OsO_4_ in ddH_2_O and 0.2 M sodium cacodylate, pH 7.3), washed 3X with ddH_2_O at room temperature, stained *en bloc* for 1 h (1% aqueous uranyl acetate) and washed again 3X with ddH_2_O. Finally, samples were dehydrated through a graded series of ethyl alcohol, passed through propylene oxide into a 1∶1 mixture of propylene oxide and Polybed embedding resin (Electron Microscopy Sciences, Hatfield, PA), followed by overnight infiltration and embedding in Polybed. Thin sections were stained with uranyl acetate and lead citrate, and photographed on a Philips 300 electron microscope operated at 60 kV. For SEM, MDA-MB-231 monolayers on serum-coated glass coverslips were treated 30 min at 37°C, fixed at 37°C (4% formaldehyde, 2.5% glutaraldehyde, 0.1 M sodium cacodylate, pH 7.4) in a laboratory microwave (Pelco, Redding, California) under vacuum for two 100 W cycles of 2 min each without changing fixative, washed 3X with buffer (0.1 M sodium cacodylate, pH 7.4) and post-fixed with buffered 1% OsO_4_. Samples were then washed 3X with ddH_2_O, microwave dehydrated at 28°C through a graded ethanol series (30, 50, 70, 80, 90, 95, 2X 100%) at atmospheric pressure for 40 s, 270 W each step, one 10 min step at RT, critically point dried (Tousimis AutoSamDri 815B), mounted to aluminum SEM stubs and sputter coated (Cressington HR208) with 8 nm gold. Images were acquired with a Hitachi S4700 FESEM.

### Transfection and infection

The EGFR-YFP plasmid was obtained from Z. Wang (University of Alberta, Edmonton, Canada). Cells were transfected with the construct using Lipofectamine 2000 (Invitrogen), replated into chamber slides, and imaged the following day on a spinning disk confocal microscope. Infection with wild type and dominant negative dynamin adenoviruses was performed exactly as described [48]. Viral stocks were a kind gift from I.R. Nabi (University of BC, Vancouver, Canada).

### Live cell imaging

EGFR-YFP-transfected MDA-MB-231 cells were plated on chamber slides (Ibidi) and maintained at 37°C and 5% CO_2_ while being imaged using a spinning disk confocal system consisting of an inverted Zeiss Axiovert 200 M microscope equipped with a QuantEM 512SC Photometrics camera. Z-stacks were obtained every 20 sec over 30 min using the 473 nm laser and a 63X objective (63X/1.4 Oil DIC Planapo).

### NeoA isolation, NMR spectroscopy and structure calculations

Purified NeoA from *Neopetrosia sp.* [15] was HPLC-purified, lyophilized, and dissolved in 4∶1 C_2_H_3_CN/H_2_O to 8 mM. The acidic pH of the sample, due to residual trifluoroacetic acid, was not adjusted. The three dimensional structure of NeoA was elucidated from natural abundance 2D ^1^H,^1^H-DQF-COSY, ^1^H,^1^H-TOCSY, ^15^N,^1^H-HSQC, ^13^C,^1^H-HSQC, ^1^H,^1^H-NOESY (τ_mix_ = 300 ms), and ^13^C,^1^H-HSQC-NOESY (τ_mix_ = 300 ms) spectra, recorded at 25°C on Varian Inova or Bruker Avance 600 MHz NMR spectrometers equipped with cryoprobes. Spectra were processed using nmrPipe [49] and analyzed using Sparky (T.D. Goddard, D.G. Kneller, Sparky 3, University of California, http://www.cgl.ucsf.edu/home/sparky/). A weak set of additional amide ^1^H^N^-^15^N resonances, attributed to disulfide bond isomerization, was observed for several residues (Phe2, Cys3, Phe5, Gly6, Cys7, Cys12, Gly13, Asn15, Arg19, Phe23, Cys26, Asp27, Cys28) in the ^15^N,^1^H-HSQC spectrum of NeoA; these were excluded during the structural analysis. Also, since the absolute stereochemistry of the methionine sulfoxide is not known, an *R*-configuration was arbitrarily set at the sulfur stereocenter. An initial ensemble averaged structure was calculated from a linear template with ARIA [50] using manually-assigned NOE-derived distance restraints in combination with distance constraints (Cβ*_i_*−Cβ*_j_*, Cβ*_i_*−Sγ*_j_*, Sγ*_i_*−Cβ*_j_*, Sγ*_i_*−Sγ*_j_*) for the previously identified Cys3−Cys26, Cys7−Cys12, and Cys18−Cys28 pairs [15]. Once approximately positioned, covalent disulfide bonds were introduced between these Cys residues, and a set of 200 structures calculated using CNS [51], from which the 24 lowest energy structures were selected to yield the final NeoA structural ensemble. A summary of the restraints and structural statistics for this ensemble is provided in Table S1 and the coordinated structures have been deposited in the Research Collaboratory for Structural Bioinformatics Protein Databank (http://www.rcsb.org/pdb/) under accession code 2JUY. Promotif [52] was used to identify secondary structure elements in the final ensemble.

## Supporting Information

Table S1 NMR and refinement statistics for NeoA.
(0.04 MB DOC)

Figure S1 NeoA prevents HT-1080 cell elongation and invasion into Matrigel. (A) HT-1080 cells were treated with NeoA (or 5 µM dihydromotuporamine C as a positive control, +ve) in constant amounts of DMSO. Cells that failed to invade were recovered and quantified through an MTT assay. Shown are averages of triplicates ± SD. **P<0.005, ***P<0.0005 compared to 0 µM as determined by two-tailed Student's t-test. (B) HT-1080 cells were plated on the reconstituted basement membrane substratum Matrigel in the presence of DMSO vehicle alone or 6.5 µM NeoA and morphology was assessed by live phase contrast microscopy after 2.5 h. Scale bar, 50 µm. (C) NeoA treatment results in loss of polarity and decreased actin ruffling. f-actin was visualized with fluorescently labeled phalloidin after HT-1080 cells had been treated with either DMSO or 3.2 µM NeoA for 90 min. Scale bar, 10 µm.
(0.81 MB TIF)

Figure S2 NeoA inhibits cellular adhesion and causes the disassembly of focal adhesions in HT-1080 cells. (A) Cells treated for 1 h with DMSO (control) or 6.5 µM NeoA were fluorescently stained for the focal adhesion proteins paxillin, vinculin, and phosphorylated focal adhesion kinase (pFAK). Note that all proteins were no longer localized at discrete attachment sites (i.e. focal adhesions) in NeoA-treated cells. Scale bar, 10 µm. (B) Adhesion to fibronectin (FN) and collagen type I (Col I) is significantly decreased in the presence of NeoA. Shown are averages of triplicates ± SD. ** P<0.05, *** P<0.005 compared to 0 µM as determined by two-tailed Student's t-test. (C) Cells pre-attached on fibronectin begin to lose adherence between 10 to 25 min after the start of treatment with 6.5 µM NeoA. Scale bar, 50 µm.
(1.84 MB TIF)

Figure S3 NeoA is slightly toxic to cells after 24 h. Cell death and apoptosis of MDA-MB-231 cells treated with the indicated concentrations of NeoA for 24 h were determined by assessing propidium iodide uptake (y axis) and Annexin V staining (x axis) by flow cytometry. Cells undergoing apoptosis stain positively for Annexin V-FITC alone and would appear in the lower right quadrant of the plots. Dead cells are PI-positive and appear in the upper quadrants, either without having undergone apoptosis (upper left quadrant) or after having undergone apoptosis (upper right quadrant).
(0.14 MB TIF)
