# Supplementary material for: Release of Membrane-Bound Vesicles and Inhibition of Tumor Cell Adhesion by the Peptide Neopetrosiamide A
Source: PLoS One. 2010 May 26;5(5):e10836. doi: 10.1371/journal.pone.0010836 (PMC2877099; doi:10.1371/journal.pone.0010836)
Supplement: Table S1 — NMR and refinement statistics for NeoA. (0.04 MB DOC) [file pone.0010836.s001.doc]

**Table S1**. NMR and refinement statistics for NeoA.

| 1. **NMR distance and dihedral constraints** | | | | | |
| --- | --- | --- | --- | --- | --- |
| Total NOEs |  | 230 | | |  |
| Intra residual (*i*–*j*=0) |  | 92 | | |  |
| Sequential (|*i*–*j*|=1) |  | 92 | | |  |
| Medium range (1<|*i*–*j*|<5) |  | 18 | | |  |
| Long range (|*i*–*j*|>4) |  | 28 | | |  |
| Total Phi dihedral constraints |  | 16 | | |  |
| 1. **Structure statistics** | | | | | |
| Violations (mean ± s.d.) | | | | | |
| Distance constraints (Å) | 0.0106 | | ± | 0.0015 | |
| Dihedral angle constraints (deg.) | 0.2011 | | ± | 0.0742 | |
| Deviations from idealized geometry | | | | | |
| Bond lengths (Å) | 0.0016 | | ± | 0.0001 | |
| Bond angles (deg.) | 0.3164 | | ± | 0.0202 | |
| Improper angles (deg.) | 0.3264 | | ± | 0.0096 | |
| R.m.s.d. from mean structure | | | | | |
| Backbone (Å) | 0.662 | | ± | 0.178 | |
| Heavy (Å) | 1.324 | | ± | 0.249 | |
