# Supplementary figures and images for: Release of Membrane-Bound Vesicles and Inhibition of Tumor Cell Adhesion by the Peptide Neopetrosiamide A
Source: PLoS One. 2010 May 26;5(5):e10836. doi: 10.1371/journal.pone.0010836 (PMC2877099; doi:10.1371/journal.pone.0010836)

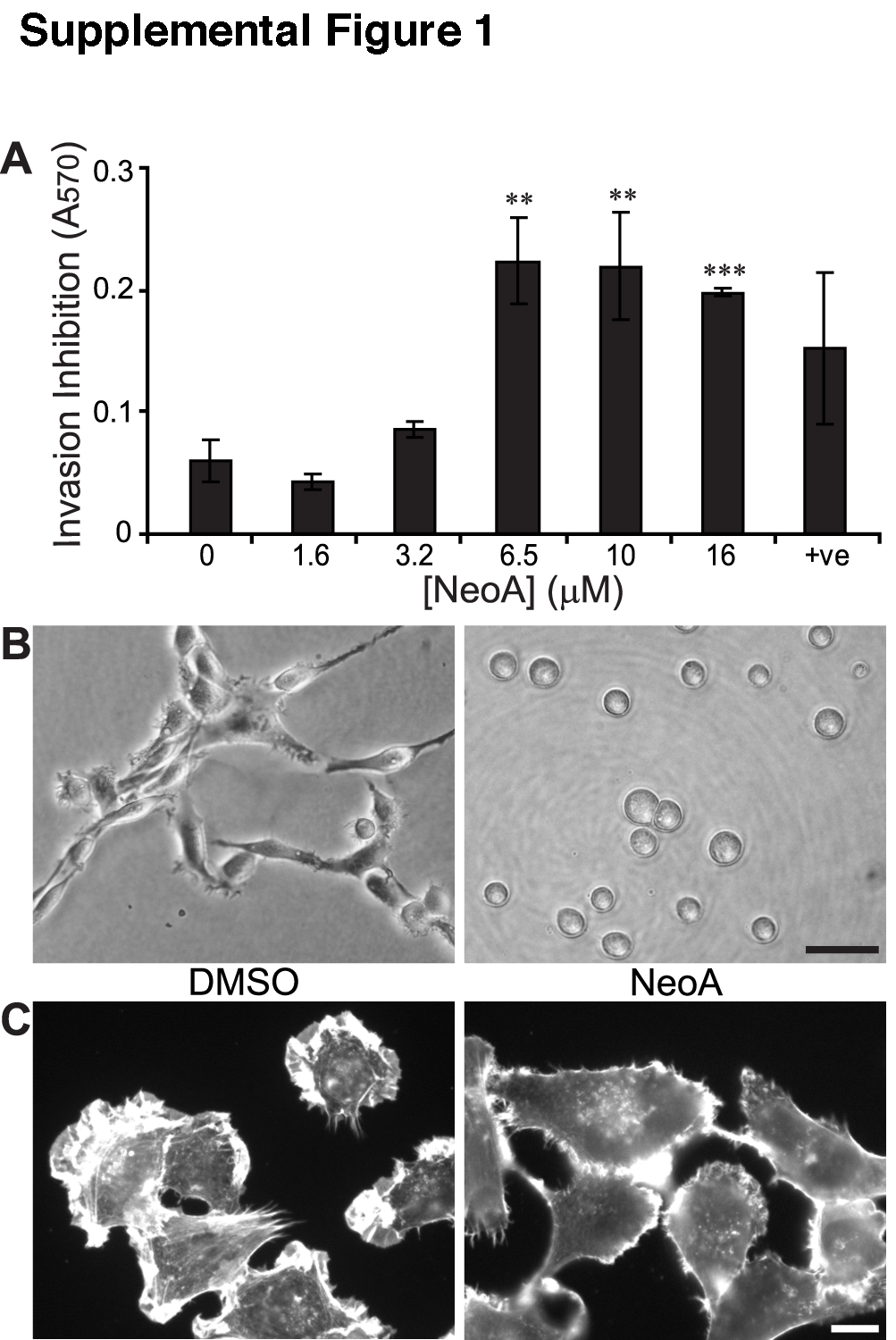

Supplement: Figure S1 — NeoA prevents HT-1080 cell elongation and invasion into Matrigel. (A) HT-1080 cells were treated with NeoA (or 5 µM dihydromotuporamine C as a positive control, +ve) in constant amounts of DMSO. Cells that failed to invade were recovered and quantified through an MTT assay. Shown are averages of triplicates ± SD. **P<0.005, ***P<0.0005 compared to 0 µM as determined by two-tailed Student's t-test. (B) HT-1080 cells were plated on the reconstituted basement membrane substratum Matrigel in the presence of DMSO vehicle alone or 6.5 µM NeoA and morphology was assessed by live phase contrast microscopy after 2.5 h. Scale bar, 50 µm. (C) NeoA treatment results in loss of polarity and decreased actin ruffling. f-actin was visualized with fluorescently labeled phalloidin after HT-1080 cells had been treated with either DMSO or 3.2 µM NeoA for 90 min. Scale bar, 10 µm. (0.81 MB TIF) [file pone.0010836.s002.tif]

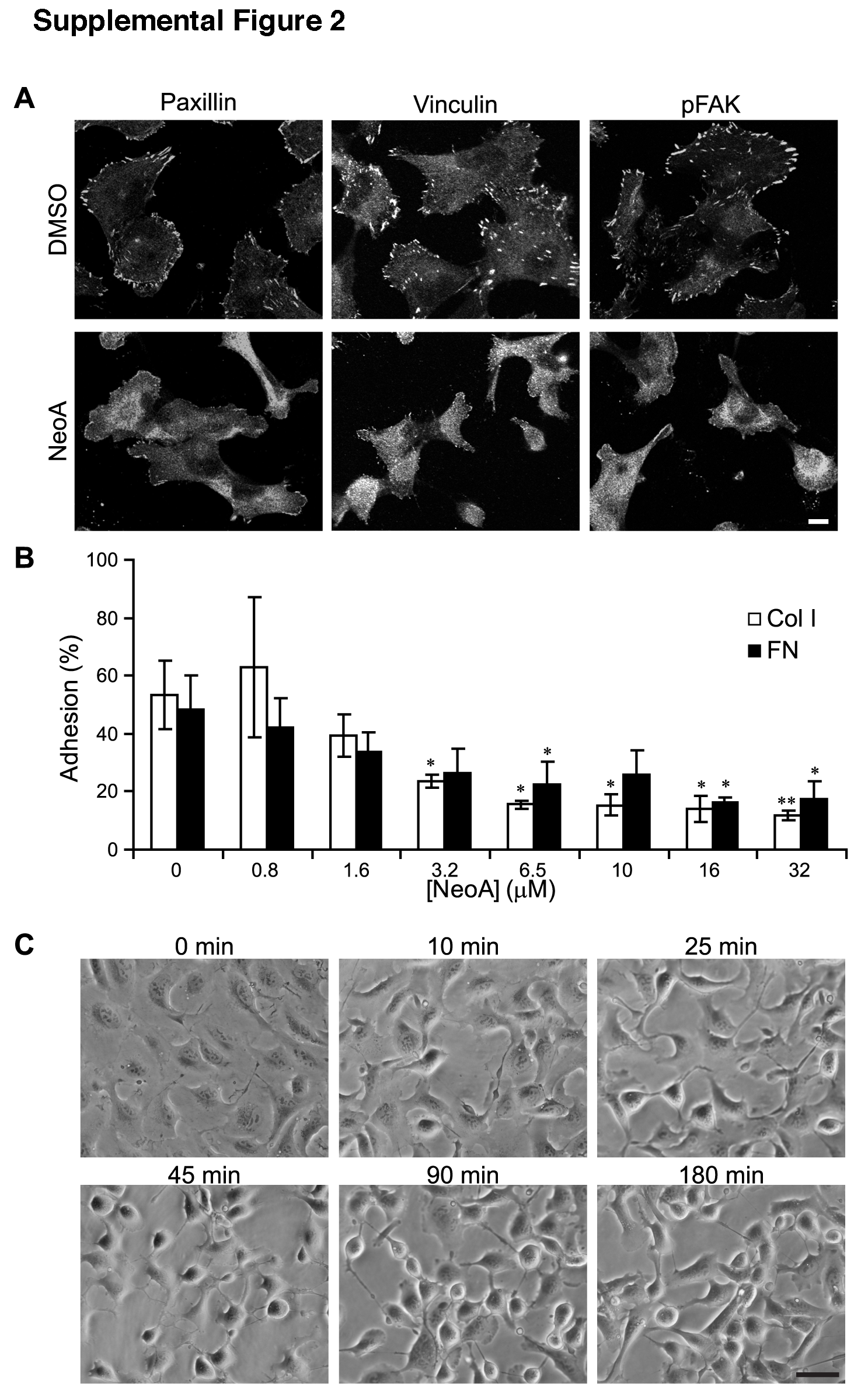

Supplement: Figure S2 — NeoA inhibits cellular adhesion and causes the disassembly of focal adhesions in HT-1080 cells. (A) Cells treated for 1 h with DMSO (control) or 6.5 µM NeoA were fluorescently stained for the focal adhesion proteins paxillin, vinculin, and phosphorylated focal adhesion kinase (pFAK). Note that all proteins were no longer localized at discrete attachment sites (i.e. focal adhesions) in NeoA-treated cells. Scale bar, 10 µm. (B) Adhesion to fibronectin (FN) and collagen type I (Col I) is significantly decreased in the presence of NeoA. Shown are averages of triplicates ± SD. ** P<0.05, *** P<0.005 compared to 0 µM as determined by two-tailed Student's t-test. (C) Cells pre-attached on fibronectin begin to lose adherence between 10 to 25 min after the start of treatment with 6.5 µM NeoA. Scale bar, 50 µm. (1.84 MB TIF) [file pone.0010836.s003.tif]

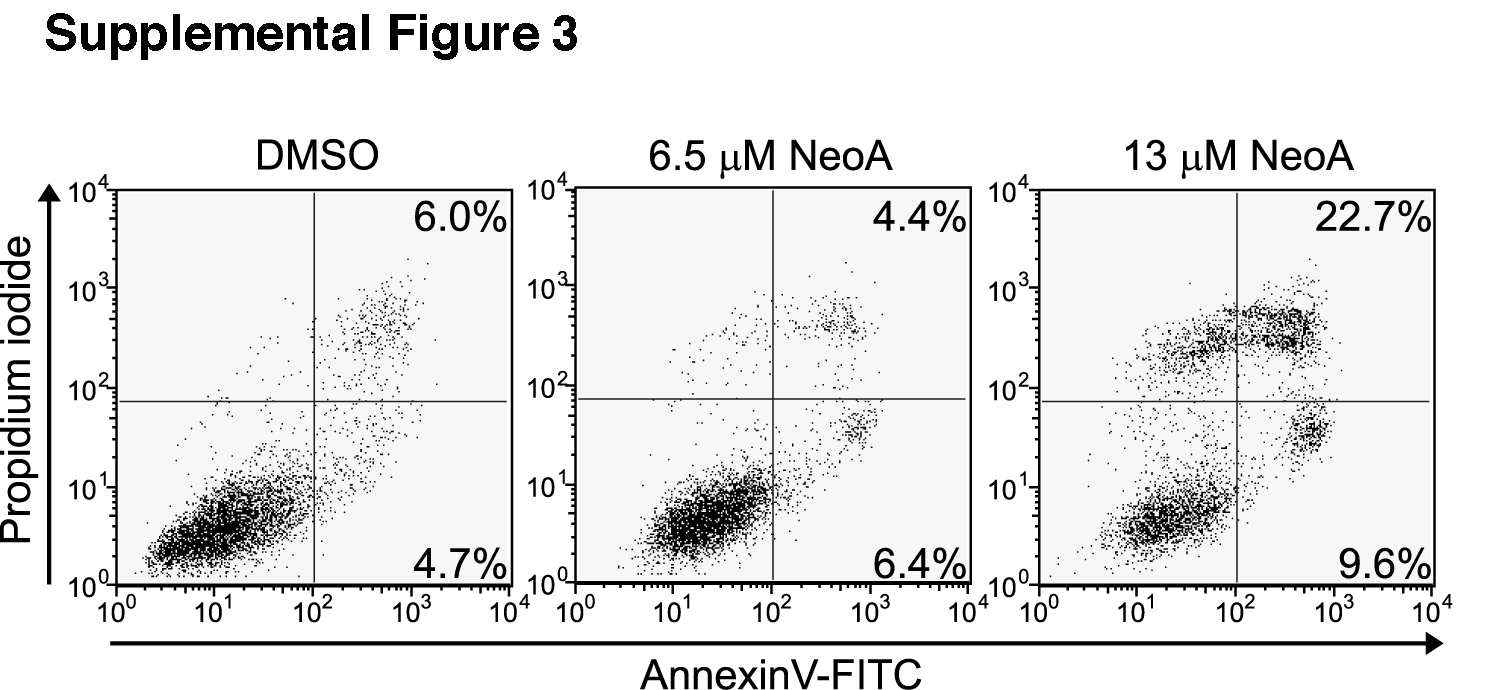

Supplement: Figure S3 — NeoA is slightly toxic to cells after 24 h. Cell death and apoptosis of MDA-MB-231 cells treated with the indicated concentrations of NeoA for 24 h were determined by assessing propidium iodide uptake (y axis) and Annexin V staining (x axis) by flow cytometry. Cells undergoing apoptosis stain positively for Annexin V-FITC alone and would appear in the lower right quadrant of the plots. Dead cells are PI-positive and appear in the upper quadrants, either without having undergone apoptosis (upper left quadrant) or after having undergone apoptosis (upper right quadrant). (0.14 MB TIF) [file pone.0010836.s004.tif]
